# The Effects of Salicyluric Acid, the Main Metabolite of Aspirin, on Lipid Peroxidation Induced by Iron and Copper Ions in a Lipid Membrane Model

**DOI:** 10.3390/ijms27031216

**Published:** 2026-01-26

**Authors:** Viktor A. Timoshnikov, Vladimir E. Koshman, Aleksandr A. Deriskiba, Nikolay E. Polyakov, George J. Kontoghiorghes

**Affiliations:** 1 Institute of Chemical Kinetics & Combustion, 630090 Novosibirsk, Russia; timoshnikov@kinetics.nsc.ru (V.A.T.);; 2 Department of Physics, Novosibirsk State University, 630090 Novosibirsk, Russia; 3 Postgraduate Research Institute of Science, Technology, Environment and Medicine, CY-3021 Limassol, Cyprus

**Keywords:** salicyluric acid, chelator, lipid peroxidation, iron complex, copper complex, molecular dynamics, ^1^H NOESY, ^1^H NMR, aspirin

## Abstract

Salicyluric acid (SUA), the main metabolite of aspirin and a natural product, is known for its ability to chelate iron and other metal ions. In particular, the chelation and increased excretion of iron by SUA may contribute to the aspirin-induced iron deficiency anemia observed in long-term aspirin users. The redox activity of iron and copper complexes of drugs and also drug metabolites, such as SUA, is an important parameter of their overall toxicity profile, including the induction of ferroptosis, which has been associated with many diseases. In this context, the effect of SUA on iron- and copper-induced lipid peroxidation and also its localization within a model lipid membrane have been investigated. A combination of physicochemical methods, including Nuclear Magnetic Resonance (^1^H NMR), molecular dynamics (MD), and Nuclear Overhauser Effect Spectroscopy (^1^H NOESY), has been used to demonstrate that SUA does not promote the peroxidation of linoleic acid micelles in the presence of Fe(II) or Cu(II) ions. NMR experiments revealed that SUA incorporates into the lipid bilayer, which stabilizes the ligands and inhibits its metal chelation ability in comparison to the control. NOESY experiments and MD simulations further showed that SUA localizes shallowly within the membrane, interacting primarily with the head group and upper acyl chain regions of lipids. These findings provide crucial insights into the membrane redox reactivity and other behavior of SUA, explaining its lack of pro-oxidant activity and also highlighting its complex role in the pharmacological and toxicological effects on iron metabolism in long-term aspirin users.

## 1. Introduction

**Scheme 1:**
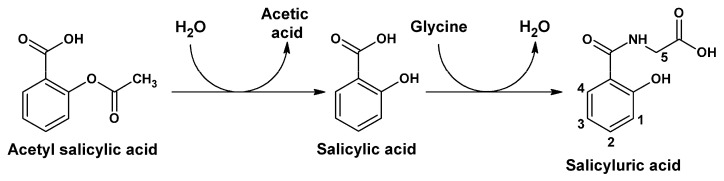
Description of the metabolic transformation of acetylsalicylic acid (aspirin) into salicylic acid and subsequently to salicyluric acid in humans. The numbers 1 to 5 indicate the position of protons in the salicyluric acid molecule.

Salicyluric acid (SUA), known also as 2-hydroxyhippuric acid, 2-[(2-hydroxybenzoyl)amino]acetic acid or N-(2-hydroxybenzoyl)glycine, is an endogenously produced metabolite and a natural product that plays a key role in the metabolism of salicylate compounds in humans, particularly aspirin [1,2,3,4]. When administered orally, acetylsalicylic acid (aspirin) is rapidly hydrolyzed to salicylic acid. Salicylic acid is subsequently conjugated with glycine in the liver to form SUA, which can account for approximately 70% of the ingested aspirin dose (Scheme 1) [5,6,7]. In addition to SUA, 2,3- and 2,5-dihydrobenzoic acids are among the major metabolites of aspirin.

**Scheme 2:**
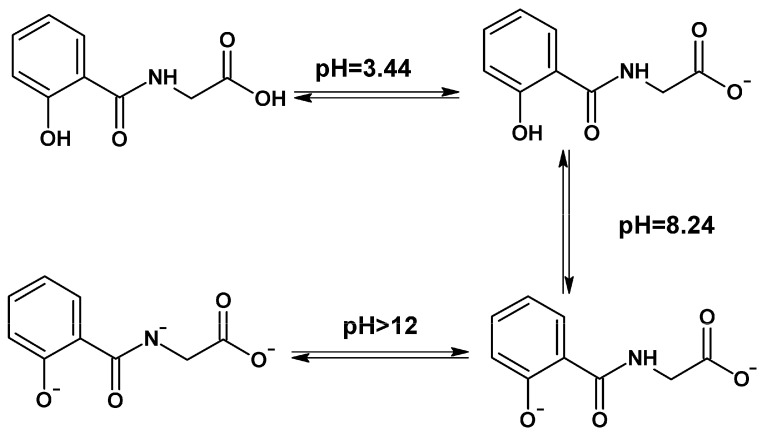
Molecular structures and electrical charge of salicyluric acid (SUA) at different pHs. At acidic pH, SUA is in a neutral form and then monoanionic (pKa_1_ = 3.44), dianionic (pKa_2_ = 8.24), and trianionic (pKa_3_ > 12) forms [8,9].

Salicyluric acid contains several functional groups, including carboxyl (-COOH), secondary amine (NH), and phenolic (-OH) groups, which enable it to exhibit a range of physicochemical and biological properties, including metal binding. Specifically, depending on the pH of an aqueous solution, SUA exists in neutral, monoanionic (pKa_1_ = 3.44), dianionic (pKa_2_ = 8.24), and trianionic (pKa_3_ > 12) forms (Scheme 2) [8,9]. Furthermore, SUA exhibits chelating activity with various metal ions, including Fe(III), Cu(II), Zn(II), and Ni(II) [8,9]. In an aqueous solution, 1:1 complexes with metal ions are predominantly formed, with a characteristic log K for Fe(III) of 2.09 [8], and for Cu(II) of 5.33 [9]. In the case of Fe(II), salicylates form unstable chelate complexes, and iron is rapidly oxidized to Fe(III) [10,11].

For more than a hundred years, the pharmacology of aspirin, including its metabolism to several metabolites, including pharmacokinetic properties and their elimination from the body, has been the focus of experimental and clinical research [2,3,12,13]. Recent studies have shown that some patients taking aspirin for long periods develop iron deficiency anemia [2,14]. One hypothesis for this relationship is the iron-chelating activity of several aspirin metabolites [2]. Salicyluric acid, as a major metabolite of aspirin, is thought to contribute significantly to this collective chelating activity.

However, this hypothesis contains numerous gaps. In particular, the effect of SUA chelation on the redox activity of complexed iron is a concern. Iron ions and iron-bound enzymes are known to be involved in a number of critical redox functions in the body, including catalytic, enzymatic, and immune functions [15,16,17]. Long-term, albeit minor, chelation of iron ions and their elimination from the body should influence the course of these processes. One such process is lipid peroxidation, initiated by hydroxyl radicals (OH•) generated during the Fenton reaction involving iron and copper ions [18,19,20,21]. Lipid peroxidation is closely linked to phenomena such as ferroptosis, a type of programmed cell death based on the disruption of cell membrane function due to its oxidation in processes involving iron ions. Numerous studies are currently underway involving aspirin and its metabolites [22,23,24,25,26], including the effect of chelation on lipid peroxidation in cells and ferroptosis [27,28,29,30,31,32,33]. Therefore, a long-term, albeit minor, impact on iron homeostasis in the body should impact the course of iron-related redox processes, particularly peroxidation. Therefore, the question remains unanswered: do aspirin metabolites, particularly SUA, influence redox processes in cell membranes?

To address this question, the following objectives were posed: first, to investigate SUA localization in the lipid bilayer, and second, to investigate the effect of SUA on lipid peroxidation. These objectives were addressed using Nuclear Magnetic Resonance spectroscopy (^1^H NMR), Nuclear Overhauser Effect Spectroscopy (^1^H NOESY) and computational methods, including quantum chemical modeling and molecular dynamics (MD). Micelles and bicelles were used as lipid membrane model systems.

The findings of this investigation may provide a more complete understanding of the role of SUA beyond its metabolic function, which is crucial for a complete picture of aspirin pharmacology and its impact on human health.

## 2. Results

### 2.1. Localization of Salicyluric Acid in the Lipid Bilayer Studied by ^1^H NOESY NMR

**Figure 1:**
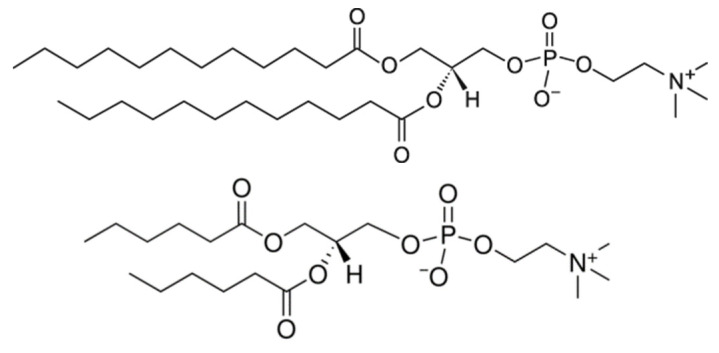
The structures of the phospholipids 1,2-dilauroyl-sn-glycero-3-phosphocholine (DLPC) and 1,2-diheptanoyl-sn-glycero-3-phosphocholine (DHPC), which were used as a membrane model.

The selective gradient NOESY (Nuclear Overhauser Effect Spectroscopy) technique was used to study the interaction of the aspirin metabolite SUA with a model lipid membrane, where 1,2-dilauroyl-sn-glycero-3-phosphocholine (DLPC)/1,2-diheptanoyl-sn-glycero-3-phosphocholine (DHPC) bicelles were used as a membrane model (Figure 1).

**Figure 2:**
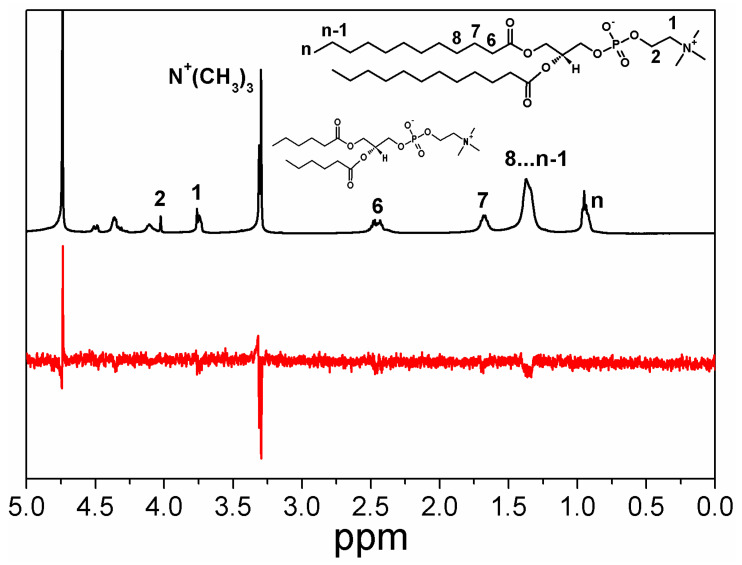
^1^H NMR (black) and selective gradient NOESY (red) spectra of 1 mM salicyluric acid (SUA) in DLPC/DHPC bicelles (PBS, pH = 7.4). Selective excitation of all SUA aromatic protons was performed. The total lipid concentration was 12 mM and the temperature T = 303 K. Symbols 1, 2, 6, 7, 8, n−1, n, N^+^(CH_3_)_3_ denote different groups of protons of the DLPC/DHPC lipids. “8…n−1” denotes the –CH_2_– signal of protons of the hydrophobic tails of DLPC/DHPC lipids from 8 to n−1.

^1^H NMR and selective NOESY (sNOESY) spectra of SUA in the bicelles are shown in Figure 2. In this method, selective excitation of aromatic protons of SUA was performed. The cross-peaks between SUA aromatic protons and lipid protons indicate the incorporation of the SUA molecule into the lipid bilayer. The signals at 4.1 and 3.7 ppm correspond to –CH_2_– protons of the hydrophilic head groups of DLPC/DHPC phospholipids. The signal at 3.29 ppm corresponds to the N–CH_3_ protons of DLPC/DHPC phospholipids. The signal at 1.36 ppm corresponds to CH_2_ protons of the hydrophobic chain of DLPC/DHPC phospholipids. The spectrum revealed an absence of NOE (Nuclear Overhauser Effect) correlations for the resonance at 0.93 ppm, assigned to the terminal methyl protons CH_3_ of the DLPC/DHPC lipids.

To quantitatively illustrate the contact probability of SUA aromatic protons with different parts of the lipid molecule, the cross relaxation rates were calculated using the following equation:

$$\sigma_{IS}=\frac{A_{IS}(t_{m})}{{t_{m}A}_{II}(t_{m})}$$

where *A_II_* is the sNOESY signal intensity of the exposed proton, *A_IS_* is the intensity of the observed cross-peak signal, and *t_m_* is the mixing time [34,35].

**Figure 3:**
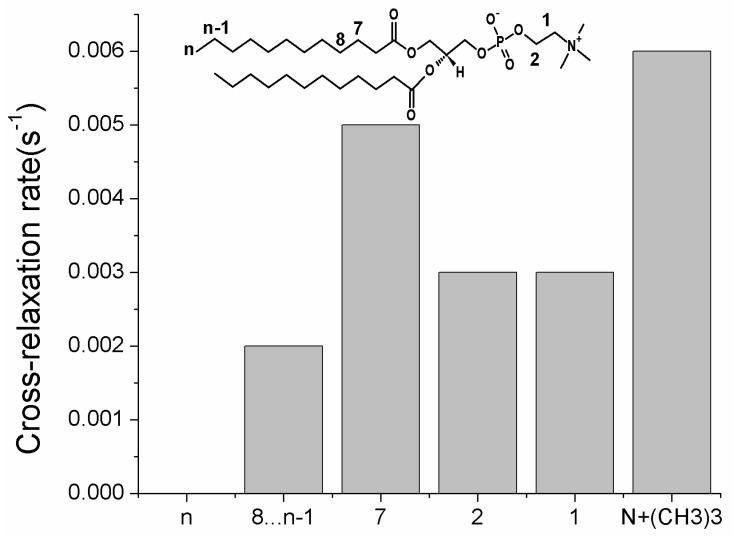
Cross-relaxation rates (s^−1^) between salicyluric acid (SUA) aromatic protons and lipid molecule segments. The total lipid concentration is 12 mM, with a salicyluric acid (SUA) concentration of 1 mM and pH = 7.4. Symbols 1, 2, 7, 8, n−1, n, N^+^(CH_3_)_3_ denote different groups of protons of the DLPC/DHPC lipids. “8…n−1” denotes the –CH_2_– signal of protons of the hydrophobic tails of DLPC/DHPC lipids from 8 to n−1.

The cross-relaxation rates for different lipid groups are presented in Figure 3. Since the cross-relaxation rate is proportional to the probability of contact with the corresponding lipid protons [35], it can be concluded that the SUA molecule can penetrate the upper regions of the lipid bilayer. However, its penetration is shallow and does not extend into the hydrophobic core, given the lack of observed cross-peaks with the terminal CH_3_ groups.

### 2.2. Calculation of Salicyluric Acid Localization in the Lipid Bilayer Using the Molecular Dynamics Method

**Figure 4:**
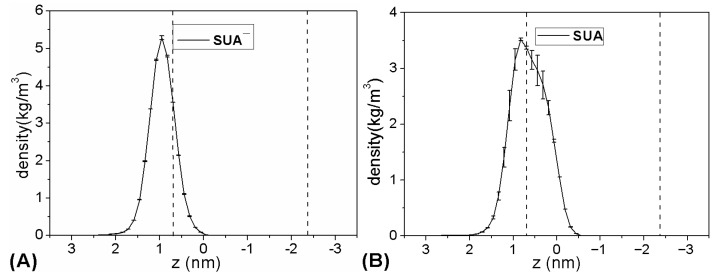
The density profiles of (**A**) deprotonated (**B**) neutral molecules of salicyluric acid (SUA) across the simulation box. The positions of DLPC N-atoms are represented by vertical dotted lines. Density profiles for both molecules were calculated as an average of two runs and represented as mean ± SD. The center of coordinates along the Z axis corresponds to the middle of the lipid bilayer.

To confirm the experimental data, MD modeling was performed. Charge distribution and bond length data were taken from quantum chemical calculations of the geometry of the SUA molecule. For the MD simulations of the ionic form, a Na^+^ counter ion was added to ensure a net-zero charge for the system. Figure 4 shows the calculated density profiles of the two forms of SUA—the monodeprotonated and the neutral forms—across the box, where the lipid bilayer is centered. The analysis reveals that the neutral form, possessing greater freedom in the membrane, penetrates more deeply into the bilayer interior, a fact clearly reflected in its density profile. The SUA molecule quickly (~1 ns) binds to the surface of the bilayer and penetrates into its interior (Supplementary Materials, Figure S1). The time to reach the membrane surface is more than an order of magnitude shorter than the run duration (600 ns), so small differences in the initial position of the molecules do not significantly affect the result.

**Figure 5:**
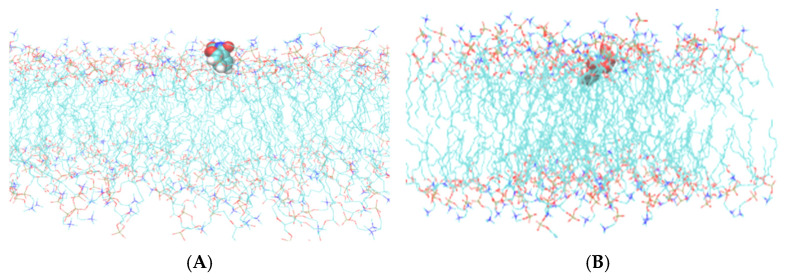
Positions of the (**A**) deprotonated and (**B**) neutral form of the salicyluric acid (SUA) molecule inside the DLPC bilayer 550 ns after starting the simulation. In case (**A**), a sodium counter ion (Na^+^) was added.

Snapshots of MD simulations of the ionic and neutral forms of SUA molecules in the lipid bilayer are given in Figure 5.

### 2.3. The Effect of Salicyluric Acid on the Peroxidation of Linoleic Acid Micelles Induced by Iron and Copper Ions Using an ^1^H NMR Method

**Figure 6:**
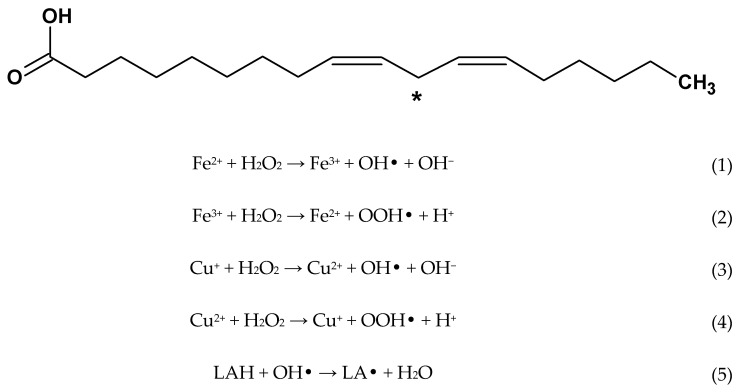
The structure of linoleic acid (LA) and the Fenton reaction, involving iron (Fe(II)) and copper (Cu(II)) ions with different changes in redox—steps (1–5). The bisallyl proton in linoleic acid is marked with an asterisk (*). (H_2_O_2_: hydrogen peroxide; OH•: hydroxyl radical; OOH•: hydroperoxyl radical; LAH: linoleic acid, containing a bisallyl proton; LA•: linoleic acid radical).

The redox activity of SUA was studied using a model of the linoleic acid (LA) micelles in peroxidation reactions induced by iron and copper ions (Figure 6). In the studies, the LA concentration was controlled by the change in the integral intensity of the bisallyl proton [36,37]. During the Fenton reaction induced by iron and copper ions, hydroxyl radicals (OH•) are formed that detach a bisallyl proton, thereby triggering an extensive chain reaction of peroxidation (initiation stage) [38,39,40,41]. The peculiarity of this reaction model (1–4) is its cyclicity, i.e., the transition between the oxidized and reduced forms of the metal ion and vice versa. The reaction continues until the hydrogen peroxide (H_2_O_2_) is fully consumed.

**Figure 7:**
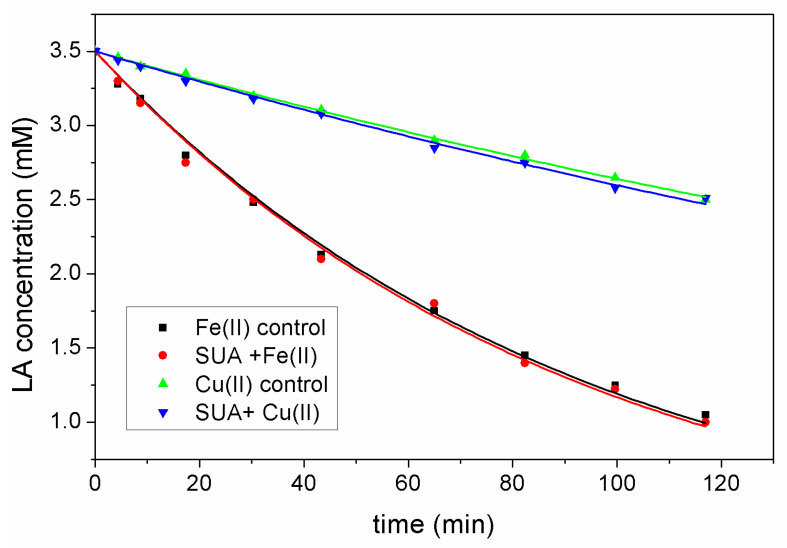
Lipid peroxidation studies in the presence of the salicyluric acid (SUA, 1 mM) and its metal complexes. The kinetic profile of the ferrous (Fe(II)) (0.1 mM) and cupric (Cu(II)) (0.1 mM) ions mediated the initiation reaction of linoleic acid (LA) (3.5 mM) peroxidation in the absence and presence of SUA at 303 °K, and pH 7.4. All experiments were performed in excess of H_2_O_2_. The points represent the experimental findings and the solid line corresponds to the exponential fitting of the points.

The time dependence of the integral intensity of the LA bisallyl signal is shown in Figure 7. It is evident that the presence of SUA has no effect on the peroxidation of LA involving iron and copper ions (Figure 7). Based on the data obtained from Figure 7, the effective rate constants for the initiation of the peroxidation of LA micelles involving iron and copper ions were calculated (Table 1).

**Figure 8:**
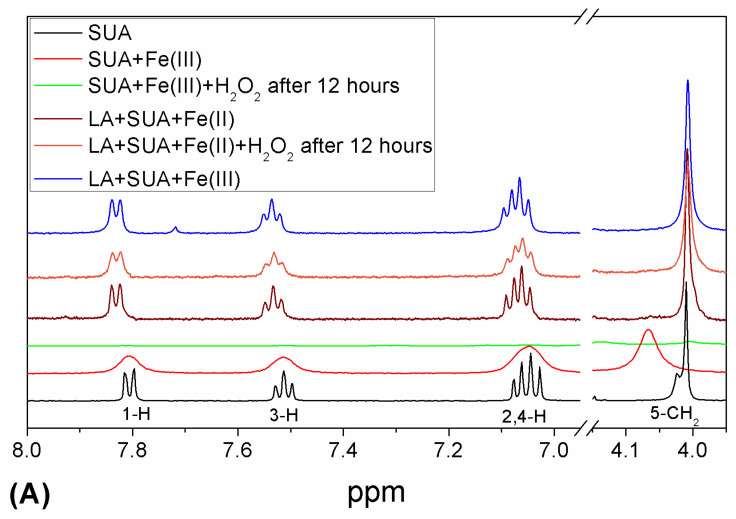

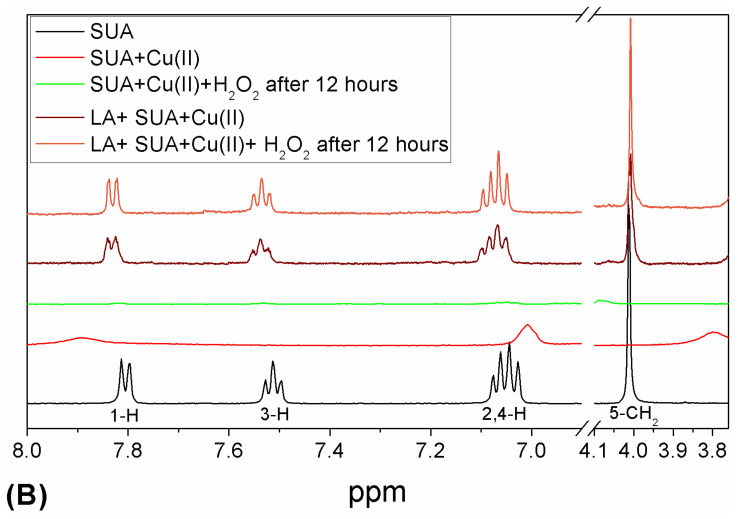
The monitoring of the interaction of salicyluric acid with iron and copper ions using Nuclear Magnetic Resonance (NMR). A section of the ^1^H NMR spectra of salicyluric acid (SUA) (1 mM) solutions with iron ions (**A**) from bottom to top as follows: SUA (1 mM) (black); SUA (1 mM) + Fe(NO_3_)_3_ (0.33 mM) (red); SUA (1 mM) + Fe(NO_3_)_3_ (0.33 mM) + H_2_O_2_ (0.5 M) after 12 h (green); LA micelle (3.5 mM) +SUA (1 mM) +FeSO_4_ (0.1 mM) +H_2_O_2_ (0.5 M) after mixing (brown) and after 12 h (orange); LA micelle (3.5 mM) + SUA (1 mM) + Fe(NO_3_)_3_ (0.1 mM). Similarly, the copper ions (**B**) are shown from bottom to top as follows: SUA (1 mM) (black); SUA (1 mM) + CuCl_2_ (0.5 mM) (red); SUA (1 mM) + CuCl_2_ (0.5 mM) + H_2_O_2_ (0.5 M) after 12 h (green); LA micelle (3.5 mM) + SUA (1 mM) CuCl_2_ (0.1 mM) + H_2_O_2_ (0.5 M) after mixing (brown) and after 12 h (orange).

To explain the lack of effect of SUA on the peroxidation of LA, a comparative analysis of the interaction of SUA proton NMR signals in aqueous phosphate-buffered saline (PBS) solutions and in micellar solutions in the presence of iron and copper ions was performed (Figure 8). It can be noted that in the presence of iron and copper ions in PBS aqueous solution, the NMR signals of the SUA protons are broadened. It is worth noting that for Cu ions, the observed effect is significantly greater than for Fe ions, and a change in the chemical shift in the SUA signals is also visible. After 12 h from the onset of redox reactions with the generation of hydroxyl radicals, the almost complete decomposition of SUA is observed in PBS aqueous solutions with both iron and copper ions. The solution becomes very turbid, and no additional signals from the decomposition products appear, indicating the formation of chelate complexes of SUA oxidation products with iron and copper ions, which are poorly soluble in aqueous media. However, under similar conditions for LA micelles, the SUA proton signals remain unchanged. A control experiment with SUA in the presence of Fe(III) ions and LA micelles showed a ^1^H NMR spectrum similar to that of SUA with Fe(II) and LA micelles, confirming the instability of the SUA-Fe(II) chelate complex and the rapid oxidation of Fe(II) to Fe(III) (Figure 8A).

## 3. Discussion

The experimental results of this study have a number of important pharmacological, biological, and physiological implications, particularly for understanding the effects of both aspirin and its metabolite SUA, on various redox processes in lipid membranes.

The results of ^1^H NOESY experiments confirmed the penetration of SUA into the lipid membrane (Figure 2 and Figure 3) and also provide information on the location of SUA based on the cross-relaxation rates. The SUA molecule was shown to interact with both the protons of the lipid heads and the protons of the tails. Further study of the location of SUA in the lipid membrane using theoretical methods, including quantum chemical calculations and molecular dynamics, showed that the density distribution profile of the monoanionic form of the SUA molecule (Figure 4A) is consistent with the experimental data obtained by the 1H NOESY method (Figure 3). For comparison, the behavior of the neutral SUA molecule in the lipid membrane was also simulated (Figure 4B). This comparison was made based on the assumption that penetration of the molecule into the lipid membrane can change the acid-base properties; in particular, pKa, which was previously demonstrated for the diclofenac molecule [42]. As a result, it was shown that the calculated density distribution profile of the neutral SUA molecule in the lipid membrane is somewhat broader than that for the SUA monoanion, but no fundamental differences in the positions of these profiles were observed. Thus, there is no clear evidence that SUA is present in the lipid membrane as a neutral molecule.

Linoleic acid peroxidation experiments showed the absence of an SUA effect on the LA peroxidation rate (Figure 7). Furthermore, the SUA penetration into the lipid membrane or micelle appears to inhibit the chelating activity of the ligands and to stabilize them in redox reactions involving transition metal ions, as confirmed by a comparison of the NMR spectra of SUA mixtures with iron and copper ions in aqueous and micellar media (Figure 8). The absence of redox activity of SUA iron and copper complexes is different from that observed for doxorubicin and other anthraquinone iron complexes, where increased lipid peroxidation and other oxidative activities occur, leading to cardiac damage [43,44,45,46,47].

**Figure 9:**
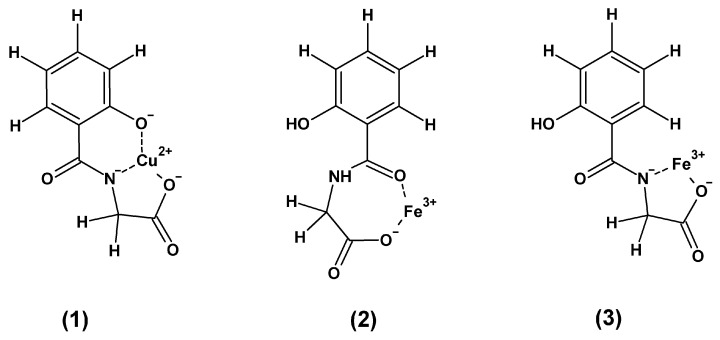
Proposed structures of chelate complexes of salicyluric acid with Cu^2+^ (**1**) and Fe^3+^ (**2**,**3**) ions formed in phosphate-buffered saline (PBS) aqueous solution of pH 7.4.

Despite the solubility limitations, the structures of the chelate complexes of SUA with copper (II) and iron (III) ions were proposed (Figure 9), following analysis of the data on the complexation of SUA with iron and copper ions [8,9], as well as the spectral changes in the ligands with these paramagnetic metal ions in water at neutral pH (7.4) (Figure 8). Previous studies have suggested that the stoichiometry of the complexes under these conditions is predominantly 1:1 [5,6]. In the case of copper ions, paramagnetic broadening of the signals in the NMR spectrum is observed, the nature of which varies for different signals of protons (Figure 8B). In addition, a change in the chemical shift (fast exchange) was detected for several groups of aromatic protons. This finding indicates primarily the coordination of the ligands and binding to the donor atom included in the aromatic structure (phenyl). Additionally, many studies indicate the affinity of copper for donor nitrogen atoms, in particular enamine, included in the chelate center [28,48,49]. On the other hand, in the case of iron ions, an identical pattern of paramagnetic broadening is observed in the NMR spectrum for each of the SUA protons (Figure 8A). Furthermore, a change in chemical shift is observed only for the CH_2_ protons. Therefore, for the iron ion, it can be suggested that the chelate center is formed by the donor atoms of the aliphatic moiety of the SUA. For comparison, the effect of chelation of iron ions with deferiprone can be described, where a difference is clearly observed between the paramagnetic effect on the aromatic and aliphatic protons of this chelator [50].

Many other factors could influence the redox and the metal-chelating activity of SUA, including steric and energetic factors, the surrounding microenvironment, interactions with other metal ions, reducing agents such as ascorbic acid, and other chelating molecules such as chelating drugs, metabolites, and nutrients [29,37,50,51,52,53,54,55].

## 4. Materials and Methods

### 4.1. Materials

Ferrous sulphate (FeSO_4_·6H_2_O, 99%), ferric nitrate (Fe(NO_3_)_3_·6H_2_O), copper chloride (CuCl_2_·2H_2_O, 99%), and H_2_O_2_ (35.5%) were obtained from Sigma-Aldrich (St. Louis, MO, USA). Linoleic acid (LA, purity > 99.0%) and salicyluric acid or 2-hydroxihippuric acid (SUA, 95%) was purchased from Shanghai Aladdin Bio-Chem Technology Co., Ltd., (Shanghai, China). The lipids 1,2-diheptanoyl-sn-glycero-3-phosphocholine (DHPC), 1,2-dilauroyl-sn-glycero-3-phosphocholine (DLPC) were purchased from Avanti Polar Lipids (Birmingham, AL, USA). All compounds were used as received. Deuterated solvent (D_2_O, 99.8% D) was obtained from Solvex-D Co. (Moscow, Russia) and was used as supplied.

### 4.2. Methods

#### 4.2.1. The ^1^H NOESY NMR Study of Salicyluric Acid Localization in a Lipid Membrane

^1^H NMR measurements were performed using a Bruker Avance HD III NMR spectrometer (operating frequency 500 MHz, Moscow, Russia, Bruker ltd). The durations of the 90° and 180° pulses were 10.9 μs and 21.8 μs, respectively. The NMR spectra were processed in the TopSpin program, version 4.0.5. All experiments were performed at a temperature of 303 K.

To study the localization of SUA in the lipid bilayer, the ^1^H NOESY NMR method was used. The description of the NMR device was given above. NOE peaks were detected by 1D selective gradient Nuclear Overhauser Effect Spectroscopy (NOESY) using the excitation sculpting technique [56]. The mixing time was optimized using a NOE build-up curve varying mixing time from 0.1 to 1 s. The mixing time in the selective gradient NOESY experiment was 0.7 s.

To prepare bicelles, the powdered components (lipids) were dissolved in chloroform, the solvent was dried, and the resulting film was hydrated with D_2_O. The chemical structures of the lipids used are shown in Figure 1. The DLPC/DHPC ratio was 1:2, with the total lipid concentration being 12 mM. The method of preparation was previously described [28].

#### 4.2.2. Theoretical Study of Salicyluric Acid Localization in a Lipid Membrane

To calculate the localization of SUA and its anion form in a lipid membrane, several software packages were used for calculating and estimating their chemical structures. Initial optimization and calculation of conformers and tautomers was performed using the CREST software package, version 3.0.2 [57,58], the semiempirical GFN-FF method [59], and an energy window of 6 kcal/mol. The solvent was taken into account during geometry optimization and conformer search using the analytical linearized Poisson–Boltzmann (ALPB) model [60]. As a result of the initial optimization, the primary structure with the lowest energy was selected.

For further geometry optimization and electron density calculation, the ORCA software package, version 5.0.3, was used [61]. Geometry optimization of the SUA molecule and its anionic form were performed using the hybrid electron density functional B3LYP and the def2-TZVP basis set [62]. In addition, an additional approximation of the Coulomb integrals was used using the def2/J basis set [63]. The Hartree–Fock exchange integrals were approximated using the chain of spheres approximation (COSX) [61]. The effect of the solvent (water) was taken into account using the polarizable continuum model (CPCM) [64]. The CHELPG (charges from electrostatic potentials using a grid-based method) method was used to calculate atomic charges [65]. The data obtained from these estimations were used for the molecular modeling of SUA in a lipid membrane.

Molecular dynamics simulations were performed to investigate the embedding of SUA into a lipid bilayer using the GROMACS 2021.4 package. The topology and atomic coordinates for the two forms of the metabolite—the neutral and the deprotonated form—were constructed in conjunction with the GROMOS54a7 force-field parameters [66]. GROMOS54a7 is widely used for MD simulations of drug–membrane interactions [67,68], and simulation results are in good agreement with experimental data on drug penetration [69,70]. For the lipid simulations, a model bilayer of DLPC (1,2-dilauroyl-sn-glycero-3-phosphocholine) introduced by Poger and Mark was utilized [71]. The simple point charge (SPC) model was used for water molecules. To maintain a net-zero charge in the system with the deprotonated form, a sodium ion (Na^+^) was added as a counter ion. Each system was initially energy-minimized using the steepest descent algorithm, followed by an equilibration simulation of 200 ps in the NVT ensemble. The production simulations were carried out in the NPT ensemble with constant pressure (1 bar) and constant temperature (T = 310 K), maintained by the semi-isotropic Parrinello–Rahman barostat [72] and the Nose–Hoover thermostat [73]. For the treatment of long-range electrostatic interactions, the Particle Mesh Ewald (PME) method was employed [74]. The initial configuration of each system consisted of a pre-equilibrated bilayer comprising 128 DLPC lipids, surrounded by water molecules (~4000), with a single molecule of the metabolite (either neutral or deprotonated) placed in the aqueous phase outside the bilayer (random position). For both the neutral and deprotonated forms, two independent production runs of 600 ns in duration were simulated. The position of the studied metabolite in the lipid membrane was analyzed using the “gmx mindist” and “gmx density” functions built into the GROMACS 2021.4 package, which allow for calculating the minimum distance between the metabolite atoms and the functional groups of lipids, as well as the density profile of the molecule along the axis perpendicular to the surface of the lipid layer, respectively. Molecules diffused rapidly to the membrane surface within about 1 ns. This time to reach the membrane is more than an order of magnitude shorter than the total production run duration, ensuring that minor differences in the initial placement of the molecules do not significantly affect the final results. For both the neutral and deprotonated forms, two independent production runs of 600 ns in duration were simulated. Density profiles for the metabolites were calculated using “gmx density” function along the Z axis (range −3.2 to 3.2 nm) in 0.1 nm steps as an average of the two runs and are represented as mean ± SD on the graphs.

#### 4.2.3. The ^1^H NMR Study of Lipid Peroxidation

The samples for the ^1^H NMR studies consisted of LA micelles, with LA concentration of 3.5 mM. In the Fenton reactions, 0.5 M H_2_O_2_ was added with 0.1 mM of FeSO_4_ or CuCl_2_ freshly prepared in phosphate-buffered saline (PBS) at pH 7.4. In order to study the effect of SUA on the oxidation of LA micelles via the Fenton reaction, the following sample preparation method was used: LA was dissolved in chloroform, dried to form a film, hydrated with PBS, and homogenized ultrasonically (1 h) to obtain micelles of uniform size. Solutions were prepared in two variants: without SUA, only the metal salt (CuCl_2_ or FeSO_4_) was added, and with SUA (final concentration 1 mM, added before the metal salt). After adding the salt, the solutions were held for 15 min, until equilibrium was reached, which was monitored by ^1^H NMR spectra.

## 5. Conclusions

The redox activity of salicyluric acid (SUA), the main metabolite of aspirin, has been studied at physiological pH in the presence of the transition metal ions iron and copper, both in phosphate-buffered saline (PBS) aqueous solution and also in a lipid membrane model of LA micelles and DLPC/DHPC bicelles. Such studies, and, in particular, drug and drug metabolite interactions with iron, may be crucial parameters of the overall toxicity profile of drugs, considering that the doxorubicin iron complex is redox-active and may cause cardiac toxicity in cancer patients, whereas SUA chelates iron ions, which may be excreted and contribute to aspirin-induced iron deficiency anemia observed in long-term aspirin users. In this context, a set of physicochemical methods, including ^1^H NMR, ^1^H NOESY, and MD, has been used to demonstrate that SUA does not influence lipid peroxidation in the presence of Fe(II) or Cu(II) ions. Furthermore, NMR experiments revealed that SUA incorporates into the lipid bilayer, which stabilizes the SUA molecules and inhibits their interaction with metal ions, in contrast with SUA behavior in PBS aqueous solutions. NOESY experiments and MD simulations have also shown that SUA localizes shallowly within the membrane, interacting primarily with the head group and upper acyl chain regions. These findings provide crucial insights into the membrane interactions of SUA, explaining its lack of pro-oxidant activity and also highlighting its complex role in the pharmacological and toxicological effects following long-term aspirin use in patients.

## Figures and Tables

**Table 1:** Effective initiation rate constants of linoleic acid peroxidation reactions induced by iron and copper ions in the absence and in the presence of salicyluric acid (SUA). The concentrations of SUA and metal salts were the same as those shown in Figure 7.

Sample	Initiation Rate Constant k, ×10^−4^ s^−1^
Control Fe(II)	10.7 ± 0.2
Control Cu(II)	2.8 ± 0.2
SUA + Fe(II)	10.9 ± 0.2
SUA + Cu(II)	3.0 ± 0.1

## Data Availability

The original contributions presented in this study are included in the article and Supplementary Materials. Further inquiries can be directed to the corresponding authors.

## Supplementary Materials

The supporting information can be downloaded at: https://www.mdpi.com/article/10.3390/ijms27031216/s1.

## Abbreviations

The following abbreviations are used in this manuscript:

DHPC	1,2-diheptanoyl-sn-glycero-3-phosphocholine
DLPC	1,2-dilauroyl-sn-glycero-3-phosphocholine
LA	Linoleic acid
MD	Molecular Dynamics
NOESY	Nuclear Overhauser Effect Spectroscopy
PBS	Phosphate-Buffered Saline
SUA	Salicyluric acid
