# Supplementary material for: The Effects of Salicyluric Acid, the Main Metabolite of Aspirin, on Lipid Peroxidation Induced by Iron and Copper Ions in a Lipid Membrane Model
Source: Int J Mol Sci. 2026 Jan 26;27(3):1216. doi: 10.3390/ijms27031216 (PMC12898443; doi:10.3390/ijms27031216)
Supplement: Supplementary file 1 [file ijms-27-01216-s001.zip › ijms-4053975-supplementary.pdf]

Supplementary materials

The Effects of Salicyluric Acid, the Main Metabolite of Aspirin, on Lipid Peroxidation Induced by Iron and Copper Ions in a Lipid Membrane Model

Viktor A. Timoshnikov [1], Vladimir E. Koshman [1], Aleksandr A. Deriskiba [1,2], Nikolay E. Polyakov [1,*] and George J. Kontoghiorghes [3,*]

[1] Institute of Chemical Kinetics & Combustion, 630090 Novosibirsk, Russia; timoshnikov@kinetics.nsc.ru (V.A.T.)
[2] Department of Physics, Novosibirsk State University, 630090 Novosibirsk, Russia
[3] Postgraduate Research Institute of Science, Technology, Environment and Medicine, CY-3021 Limassol, Cyprus
* Correspondence: polyakov@kinetics.nsc.ru (N.E.P.); kontoghiorghes.g.j@pri.ac.cy (G.J.K.)

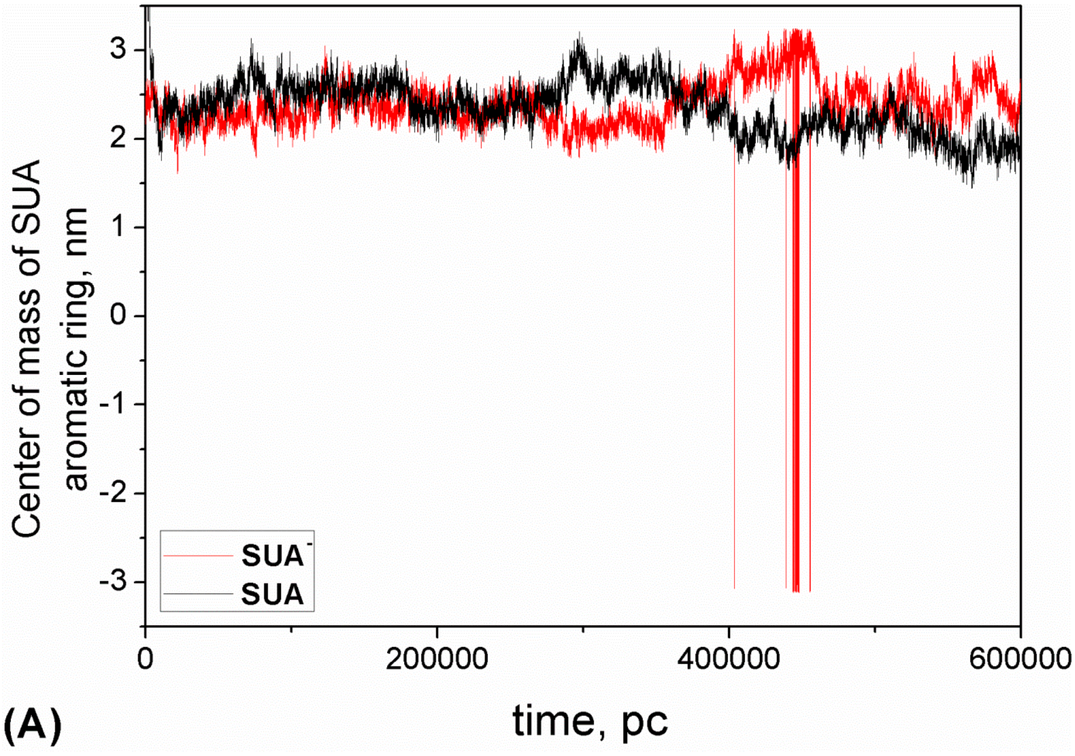

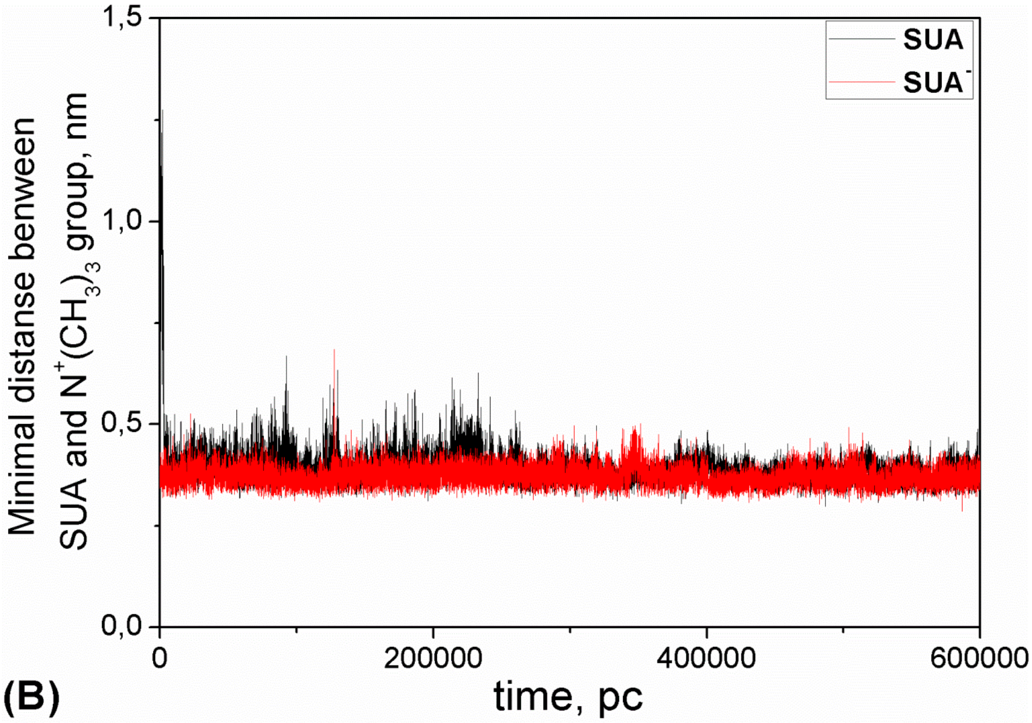

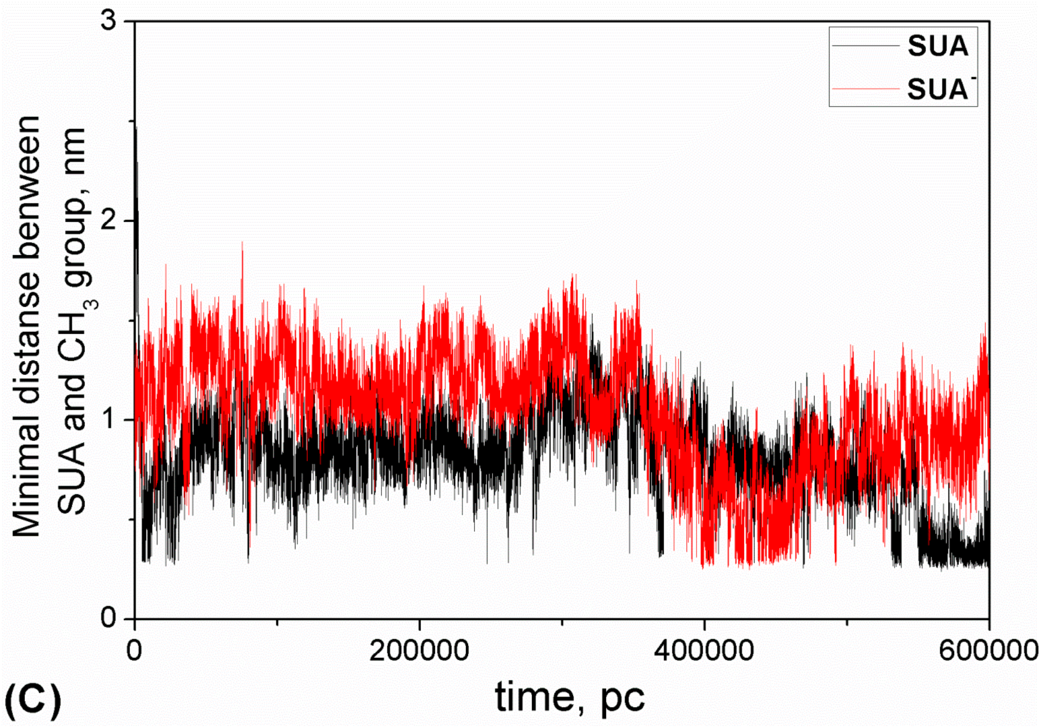

Figure S1. **(A)** – Calculated coordinate of the center of mass of the aromatic ring of SUA in the neutral and monoanionic forms along the Z axis over time. **(B)** Calculated minimum distance between SUA atoms and $N^+(CH_3)_3$ groups of lipids over time; **(C)** Minimum distance between SUA atoms and terminal CH_3 groups of lipids over time;
